# Short-term effects of exercise therapy on pulmonary function and exercise tolerance in patients with mild to moderate adolescent idiopathic scoliosis: a meta-analysis of randomized controlled trial

**DOI:** 10.3389/fspor.2026.1804651

**Published:** 2026-06-17

**Authors:** Yongqi Xie, Shaodong Xie, Jiajia Yang, Qiuhua Yu, Guifang Zhang, Yuhen Feng, Jiamin Liu, Changqing Ye, Han Gong, Chuhuai Wang, Fen He

**Affiliations:** 1 Department of Rehabilitation Medicine, Foshan Hospital of Traditional Chinese Medicine, The Eighth Clinical Medical College of Guangzhou University of Chinese Medicine, Foshan, China; 2 Department of Rehabilitation Medicine, The First Affiliated Hospital, Sun Yat-Sen University, Guangzhou, China; 3 Beijing Key Laboratory for Biomaterials and Neural Regeneration, National Medical Innovation Platform for Industry-Education Integration in Advanced Medical Devices, School of Biological Science and Medical Engineering, Beihang University, Beijing, China

**Keywords:** adolescent, exercise therapy, idiopathic scoliosis, meta-analysis, pulmonary function, randomized controlled trials

## Abstract

**Objective:**

This meta-analysis aimed to evaluate the effects of exercise therapy on pulmonary function and exercise tolerance in patients with mild to moderate adolescent idiopathic scoliosis (AIS).

**Methods:**

We systematically searched PubMed, Embase, Medline, Web of Science, and Cochrane Library from inception to April 1st, 2025. We included randomized controlled trials (RCTs) enrolling participants with mild to moderate AIS (Cobb angle 10–40°). Data were extracted for the following outcomes: forced expired volume in 1 s (FEV_1_), forced vital capacity (FVC), FEV₁/FVC ratio, peak expiratory flow (PEF), maximal expiratory pressure (MEP), maximal inspiratory pressure (MIP), and the six-minute walking test (6MWT). The Cochrane systematic review tool was used to assess the methodological quality of the RCTs, and the GRADE approach was applied to evaluate the quality of evidence.

**Results:**

Of 912 screened records, six RCTs involving 224 patients were included. All studies were judged to have a low risk of bias. Compared with control, exercise therapy improved FEV_1_ (MD = 0.41 L, 95% CI: 0.22 to 0.59, *P* < 0.001), FVC (SMD = 0.40 L, 95% CI: 0.02 to 0.78, *P* < 0.01), FEV1/FVC ratio compared to the control interventions (SMD = 0.46, 95% CI: 0.05 to 0.86, I2: 48%, *P* < 0.05), MEP (MD = 10.70 cmH₂O, 95% CI: 3.27 to 18.13, *P* < 0.01), and MIP (MD = 16.44 cmH₂O, 95% CI: 6.87 to 26.01, *P* < 0.001). Although the subgroup sample size is limited and needs to be interpreted with caution, preliminary results show that long-term (more than 8 weeks) interventions have significant improvement effects on both FEV_1_ and FVC, while a single long-term (more than 60 min) intervention only shows significant benefits on FEV_1_.

**Conclusions:**

Exercise therapy can effectively improve lung volumes and enhance respiratory muscle strength in patients with AIS in short term. Therefore, further high-quality RCTs are warranted to confirm these findings.

**Systematic Review Registration:**

PROSPERO CRD420251034100.

## 1 Introduction

Idiopathic scoliosis (IS) is the most common type of scoliosis, accounting for approximately 80% of all cases, with adolescent idiopathic scoliosis (AIS) being its most common subtype (1). Idiopathic scoliosis (IS) is prone to occur during periods of rapid growth, including the juvenile growth spurt (5–8 years of age) and the adolescent growth spurt (11–14 years of age), with the adolescent period being the most critical phase for AIS (1). This condition affects 2%–3% of adolescents globally, with incidence rates varying by geographical latitude (2, 3). In China, the reported prevalence of scoliosis is 1.2%, with mild to moderate disease severity comprising 79.10% and 16.80% of cases, respectively (4).

AIS is a musculoskeletal disorder characterized by a three-dimensional deviation of the spine, including coronal plane displacement and vertebral rotation. These structural abnormalities can alter thoracic configuration, potentially leading to respiratory dysfunction. Common pulmonary manifestations in AIS include reduced lung volumes, diminished respiratory muscle strength, and impaired exercise tolerance (5). The severity of respiratory dysfunction is influenced by multiple factors, including the degree of scoliosis, vertebral rotation, thoracic kyphosis, lordosis, and the degree of chest deformation (6). In patients with moderate (Cobb angle: 20–40°) to severe (Cobb angle >40°) curves, a significant negative linear correlation has been established between curvature severity and forced vital capacity (FVC) (7). Even patients with mild scoliosis may exhibit respiratory dysfunction, characterized by abnormal respiratory patterns, respiratory muscle impairment, and reduced exercise capacity (8, 9). Furthermore, a meta-regression analysis predicted a 1% decrease in pulmonary function for every 2.6 to 4.5 degrees in thoracic Cobb angle [forced expired volume in 1 s (FEV_1_), FVC, and total lung capacity] (10).

The treatment strategies for AIS are guided by its functional and physiological impairment (11). Common conservative treatment aimed at limiting curve progression and correcting postural deviation included bracing and exercise therapy. Bracing interventions included rigid braces and flexible scoliotic braces (12). Exercise therapy included physiotherapeutic scoliosis-specific exercise (PSSE) (13), aerobic exercise, and respiratory training (14). It remains unclear whether any form of active exercise is more beneficial for AIS patients than traditional rehabilitation. In addition, determining a clear exercise prescription, particularly the time and frequency required to effectively improve lung function, is a key priority in this field. A previous meta-analysis reported positive effects of exercise training on FVC and FEV_1_ in patients with AIS (14). And it only included a limited number of literature and the research types included randomized controlled trials (RCTs) and cohort studies. Currently, there is a lack of meta-analyses that exclusively focus on RCTs investigating exercise interventions, especially in populations with mild to moderate AIS.

Therefore, this systematic review aimed to synthesize evidence exclusively from RCTs to evaluate the efficacy of exercise therapy and control group (general physical therapy) on pulmonary capacity parameters and exercise capacity. Secondly, we will explore the sources of heterogeneity through subgroup analysis based on exercise type, intervention duration. Finally, we will assess the quality of evidence to provide information for clinical practice and guide future research directions.

## 2 Methods

This systematic review and meta-analysis was reported following the guidelines for Preferred Reporting Items for Systematic reviews and Meta-analyses (PRISMA) (15). The protocol was registered with the Prospective Register of Systematic Reviews (PROSPERO) with registration number CRD420251034100.

### 2.1 Search strategy

The review was searched in the following five databases: PubMed, Web of Science, Scopus, Embase, and the Cochrane Library. We included all articles published in English from database inception until April 1st, 2025, with no restrictions on the country of origin. The search strategy was built using key terms related to “scoliosis”, “exercise therapy”, and “randomized controlled trials”, including Medical Subject Headings (MeSH) terms and relevant keywords. The strategy for all databases is detailed in Supplementary Material 1 (Supplementary Text File 1). Mild to moderate scoliosis was defined as a Cobb angle between 10°and 40°.

### 2.2 Study selection

The population, intervention, comparison, outcome, and studies (PICOs) framework was used to retrieve and screen articles. The inclusion criteria for the meta-analysis were as follows: (1) males and females aged between 6 and 18 years old diagnosed with AIS (a Cobb angle between 10°and 40°). (2) The study compared an exercise intervention (e.g., PSSE, aerobic exercise, and respiratory training) against a control group (e.g., usual care, general physical therapy). Studies comparing different modes of intervention (e.g., supervised vs. unsupervised) were also considered eligible, as they provide valuable information on the effectiveness of exercise interventions. (3) The study was designed as a RCT and reported at least one pulmonary function outcome.

The exclusion criteria were as follows: (1) Non-RCT designs (e.g., case-control studies, case reports, conference papers, systematic reviews, and other research), (2) intervention group does not include spine surgery or braces, (3) duplicate publications, (4) irrelevant populations or interventions, (5) full text unobtainable, and (6) post-surgical exercise interventions.

### 2.3 Primary and secondary outcomes

The primary outcomes were FEV_1_ and FVC. Secondary outcomes included the FEV_1_/FVC ratio, peak expiratory flow (PEF), maximal inspiratory pressure (MIP), maximal expiratory pressure (MEP), and the 6-minute walking test (6MWT).

### 2.4 Data extraction

Two researchers (YX and HG) independently performed the study selection process using a literature manager (EndNote X9, Philadelphia, PA). Disagreements were resolved by the third researcher.

Study characteristics were extracted from the included studies: 1) basic information: first author, year of publication, country, 2) characteristics of the study population: age, gender, and scoliosis curve, 3) outcome measures, and 4) intervention details: intervention type, sessions, duration, and devices.

### 2.5 Quality assessment

The methodological quality of the included studies was assessed by the Cochrane RoB tool (16) and the physiotherapy evidence database scale (PEDro) (17). Two researchers conducted the quality assessment independently. The Cochrane RoB tool evaluates seven domains: random sequences, allocation concealment, blinding of participants and related personnel, blinding of assessors, incomplete outcome data, selective reporting, and other sources of bias. For each study, each criterion was scored as “low,” “high,” or “unclear risk”. Disagreements were resolved by consensus or by arbitration from a third reviewer. The PEDro scale is an 11-item scale and is not used to calculate the total score. In addition, 7–10 points are classified as high quality literature, 5–6 points are classified as medium quality literature, and ≤4 points are classified as low quality literature (18).

The certainty of the evidence for each outcome was evaluated using the GRADE (Recommended Assessment, Development, and Evaluation Grading) approach. The evidence was rated as “high,” “moderate,” “low,” or “very low,” based on considerations of risk of bias, inconsistency, inaccuracy, indirectness, and publication bias.

### 2.6 Data analysis

Statistical analyses were performed using Review Manager software (Version 5.4). For continuous outcomes, we calculated the mean difference (MD) and standard deviation (SD) in two groups. When outcomes were measured using consistent units across studies, they were synthesized using MD and 95% confidence interval (CI). The standardized mean difference (SMD) was used for outcomes measured on different scales (e.g., FVC and FEV_1_/FVC). The overall effect sizes (MD or SMD) and their 95% CI were computed to compare the intervention and control groups. A two-sided *P*-value of less than 0.05 was considered statistically significant.

### 2.7 Heterogeneity and sensitivity analysis

The heterogeneity among the included research results was analyzed using the Chi-square test, and the magnitude of heterogeneity was quantitatively determined by combining the *I^2^* statistic. *I^2^* values of 25%, 50%, and 75% were interpreted as indicating low, moderate, and high heterogeneity, respectively. The *I^2^* statistic was explained as follows: statistical significance was considered when the *P* value was less than 0.05 and the *I^2^* statistic was greater than 50%. A random-effects model was employed for meta-analysis to account for potential clinical and methodological heterogeneity.

Sensitivity analyses were conducted to examine the robustness of the results by sequentially excluding each study and visually inspecting funnel plots to assess potential publication bias.

## 3 Results

### 3.1 Study selection and characteristics

**Figure 1:**
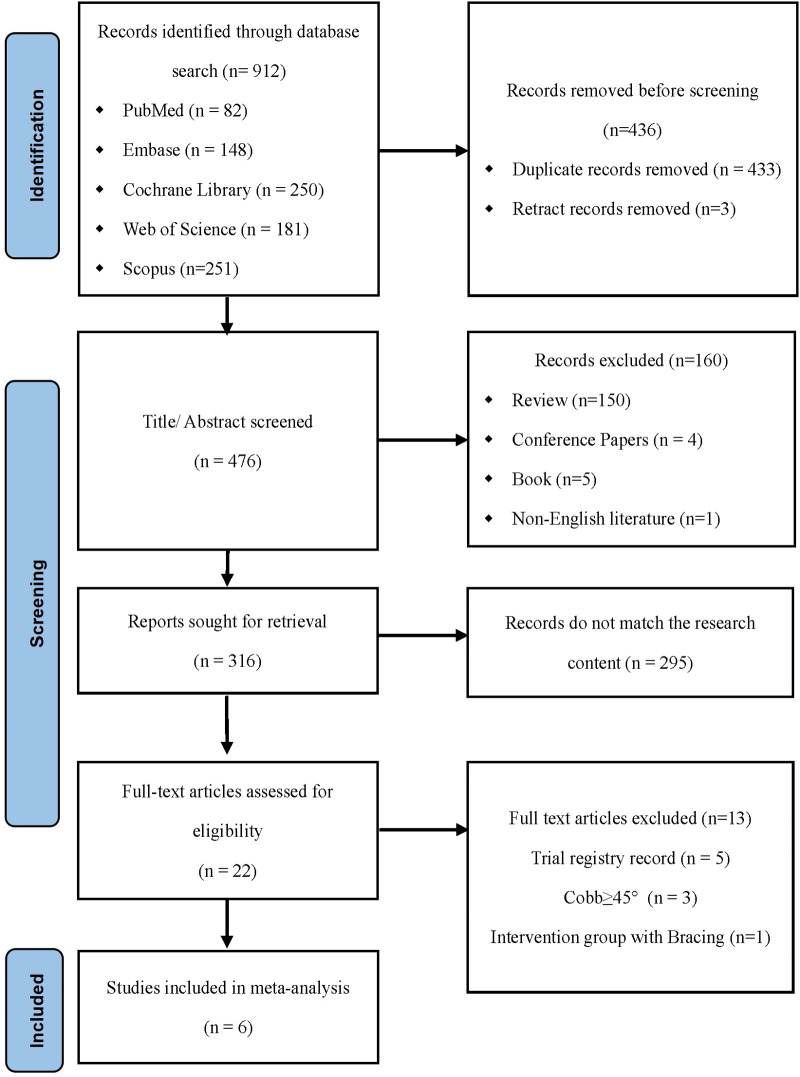
PRISMA flowchart diagram of the study. Adapted from the PRISMA 2020 flow diagram template by Page et al. (33). This work is licensed under CC BY 4.0. To view a copy of this license, visit https://creativecommons.org/licenses/by/4.0/. Modified by the authors.

A total of 912 studies were retrieved using the above retrieval strategies. Duplicate literature (*n* = 433) was excluded. The study selection process is detailed in the PRISMA flow diagram (Figure 1). The basic characteristics of the included studies are summarized in Table 1. The six RCTs enrolled a total of 224 patients with AIS, including 183 females (81.7%) and 41 males (18.3%). These studies were conducted in six countries: Brazil (13), China (19), Turkey (20), India (21), Saudi Arabia (22), and Serbia (23). The average baseline age of the participants ranged from 10.10 to 19.33 years. The Cobb curve averages between 12°−30°.

**Table 1:** Patient characteristics of the included studies.

Author year	Country	Total sample size (M/F)	Group	Gender (M/F)	Age (years)	Curve	Measures
Kumar, 2017 (21)	India	36 (21/15)	I	11/7	12.17 ± 1.72	12.61 ± 1.81	①②③④
			C	10/8	11.56 ± 1.46	12.72 ± 1.40	
Gao, 2019 (19)	China	45 (9/36)	I	5/18	12.22 ± 1.35	28.64 ± 3.91	①②③
			C	4/18	12.14 ± 1.32	29.13 ± 4.32	
Abdel Ghafar, 2022 (22)	Saudi Arabia	45 (17/28)	I	9/13	14.68 ± 1.72	15.59 ± 2.66	①②③⑦
			C	8/15	15.04 ± 1.66	16.32 ± 2.39	
Yildirim, 2022 (20)	Turkey	30 (4/26)	I	2/13	13.80 ± 2.85	18.80 ± 6.53*	②④⑤⑦
			C	2/13	15.87 ± 3.46	22.00 ± 7.58*	
Basbug, 2023 (13)	Brazil	34 (0/34)	I	0/17	13.70 ± 1.80	21.20 ± 11.60*	①②④⑥⑤⑦
			C	0/17	13.90 ± 1.83	24.20 ± 11.0*	
Dimitrijević, 2025 (23)	Serbia	34 (10/24)	I	5/12	14.11 ± 1.02	30.18 ± 8.19	①②③
			C	5/12	13.41 ± 1.63	30.24 ± 6.51	

M, Male; F, Female; I, Intervention group; C, Control group. ① FEV_1_, ② FVC, ③ FEV_1_/FVC, ④PEF, ⑤MEP, ⑥MIP, ⑦ 6MWT.

* Lumbar Cobb.

The exercise intervention included PSSE, core stabilization training, breathing exercises, and health management. One study applied task-oriented exercises (21), one applied hippotherapy and PSSE (22), one used core stabilization training (20), one used PSSE (23), two studies applied breathing exercises and other exercises (13, 19). In the control group, one study applied bracing (19), which was adjusted every 3 months. The intervention duration per session ranged from 15 to 90 min, and the total program length varied from 8 to 52 weeks. The specific exercise intervention methods are shown in Table 2.

**Table 2:** Intervention group and control group information.

Author year	Group	Intervention	Sessions	Duration (wk)	Device
Kumar, 2017 (21)	I	Task oriented exercises	65mins/session	52	-
		Spinal strengthening exercises			
		Spinal extension			
		Breathing exercises			
		Active self-correction			
	C	Spinal strengthening exercises	40mins/session		
		Spinal extension			
		Breathing exercises			
		Active self-correction			
Gao, 2019 (19)	I	Breathing exercises	40mins/wk	24	Spirometry (CareFusion,Germany)
		Active self-correction			
	C	Brace (TLSO)	23 h/day		
Abdel Ghafar, 2022 (22)	I	Hippotherapy	Hippotherapy: 30mins/wk	10	Spirometer (Spiro Master PC-10, Chest M.I.Inc, Japan)
		Schroth exercises			
		Active self-correction	60mins/three times per wk		
	C	Schroth exercises	60mins/ three times per wk		
		Active self-correction			
Yildirim, 2022 (20)	I	Core stabilization training	60mins/day/wk	8	Spirometry (Cosmed Pony FX, Italy)
		Traditional scoliosis exercises			
	C	Home exercises	60mins/day/wk		
		Traditional scoliosis exercises			
Basbug, 2023 (13)	I	Inspiratory muscle training	IMT: 15mins/bid/day	8	Threshold IMT device® (Philips, Amsterdam,Netherlands)
		Home exercises	45mins/session/wk		
		Spinal stabilization exercises			
		Spinal extension			
		Active self-correction			
	C	Home exercises	45mins/session/wk		
		Spinal stabilization exercises			
		Spinal extension			
		Active self-correction			
Dimitrijević, 2025 (23)	I	Schroth exercises under a physiotherapist	90mins/three times per wk	8	Spirometer (MIR,Rome, Italy)
	C	Schroth exercises independently	-		

I, Intervention group; C, Control group.

### 3.2 Risk of bias

**Figure 2:**
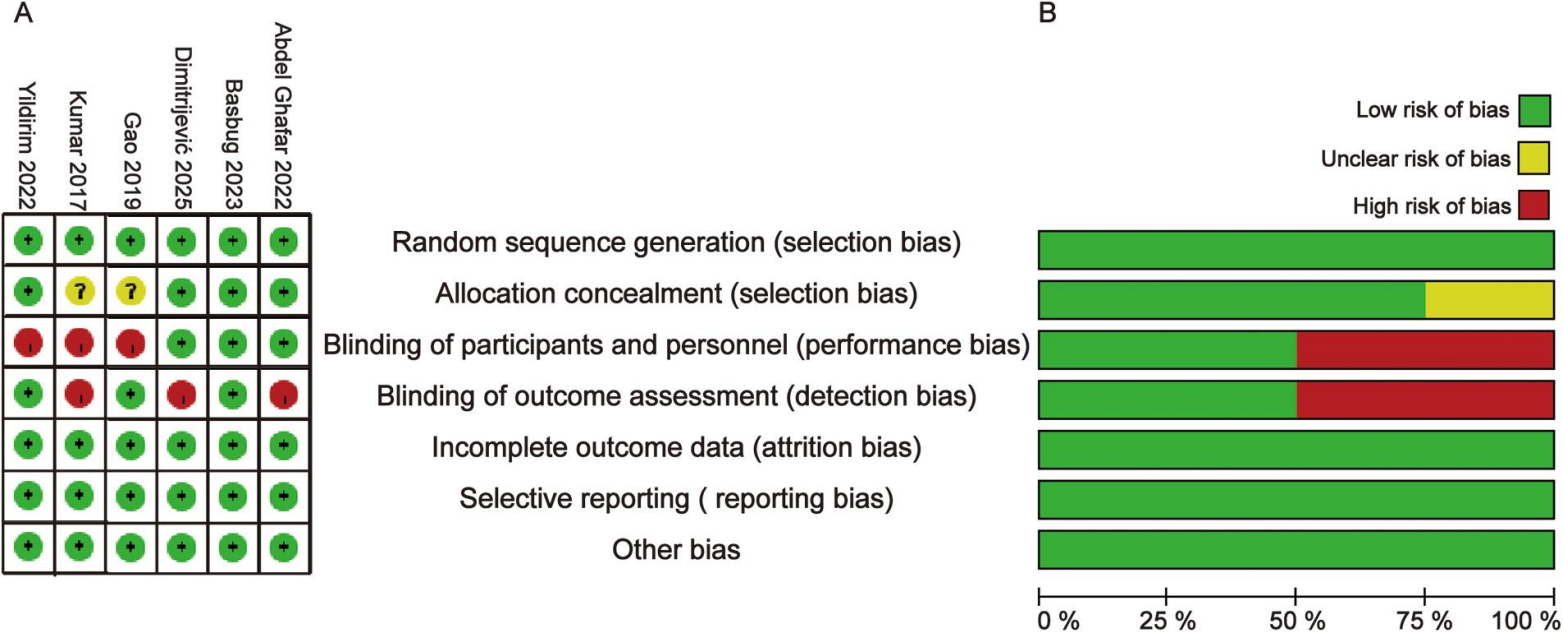
Risk of bias by the cochrane tool.

Figure 2 displays the risk of bias for the included RCTs assessed using the Cochrane tool. Four studies (13, 20, 21, 23) used a double blind method, two studies (19, 22) used a single blind method. Six studies described adequate random sequence generation (13, 19–23). According to the PEDro scale, there were six high-quality studies (Table 3).

**Table 3:** Summary of PEDro scale.

Inclusion study	1	2	3	4	5	6	7	8	9	10	11	Total
Kumar, 2017 (21)	✓	✓	✓	✓	✓		✓	✓	✓	✓	✓	10
Gao, 2019 (19)	✓	✓	✓	✓			✓	✓	✓	✓	✓	9
Abdel Ghafar, 2022 (22)	✓	✓	✓	✓	✓			✓	✓	✓	✓	9
Yildirim022 (20)	✓	✓	✓	✓	✓		✓	✓	✓	✓	✓	10
Basbug, 2023 (13)	✓	✓	✓	✓	✓		✓	✓	✓	✓	✓	10
Dimitrijević, 2025 (23)	✓	✓	✓	✓	✓		✓	✓	✓	✓	✓	10

1, eligibility criteria; 2, randomly allocated; 3, assigning concealment; 4, similar at baseline; 5, blinding of all subjects; 6, blinding of all therapists; 7, blinding of all assessors; 8, measures of at least one key outcome; 9, intention to treat; 10, comparison between groups; 11, point measures and measures of variability.

### 3.3 Quality of the evidence

The overall quality of the evidence, as evaluated by the GRADE approach, ranged from moderate to low for the various outcomes. This was primarily due to concerns regarding heterogeneity and the small population size in the included studies. The summary of findings, including the quality of the evidence and the effect estimates of all the outcomes, is shown in Supplementary Material 2.

### 3.4 Primary outcomes

#### 3.4.1 FEV_1_

**Figure 3:**
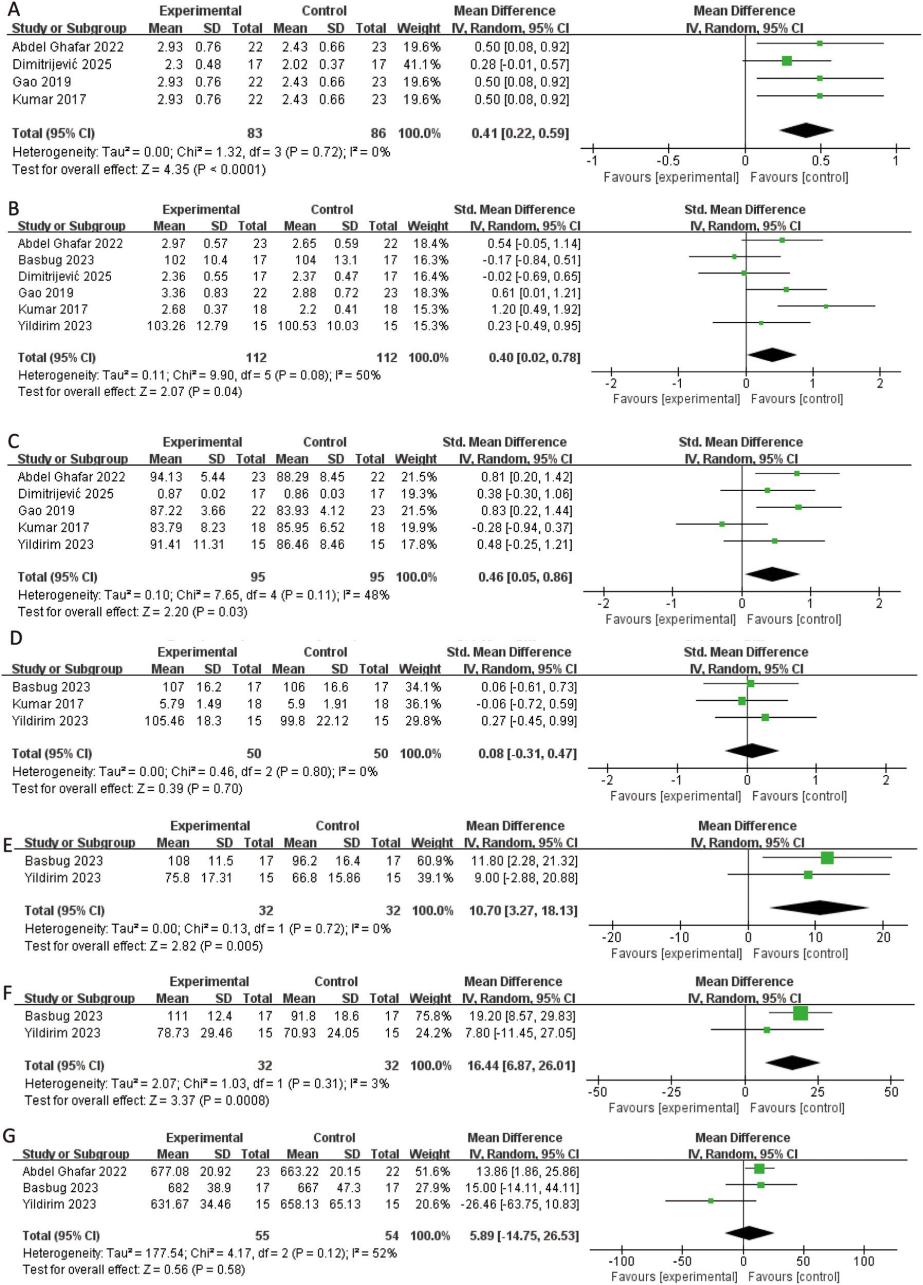
Forest plot of exercise therapy intervention on pulmonary function indicators in AIS patients in randomized controlled trials. A) forced expired volume in 1 s (FEV1), B) forced vital capacity (FVC), C) FEV1/FVC, D) peak expiratory flow, E) maximal expiratory pressure (MEP), F) maximal inspiratory pressure (MIP), G) six-minute walking test (6MWT).

Four studies involving 169 participants reported data on FEV_1_ (19, 21–23) and were included in the quantitative synthesis. The meta-analysis demonstrated that exercise therapy produced a significant improvement in FEV_1_ compared to the conservative treatments (MD = 0.41, 95% CI: 0.22 to 0.59, I^2^: 0%, *P* < 0.0001) (Figure 3A).

#### 3.4.2 FVC

All seven studies, encompassing 224 participants, reported FVC outcomes (13, 19–23). The meta-analysis revealed a significant benefit on FVC of exercise therapy over conservative treatment (SMD = 0.40, 95% CI: 0.02 to 0.78, I^2^: 50%, *P* < 0.05) (Figure 3B).

### 3.5 Secondary outcomes

#### 3.5.1 FEV_1_/FVC

There were five studies (*n* = 190) that compared the effects of exercise therapy to other therapies on FEV_1_/FVC (19–23). The meta-analysis showed that exercise therapy significantly improved the FEV_1_/FVC compared to the control interventions (SMD = 0.46, 95% CI: 0.05 to 0.86, I^2^: 48%, *P*  <  0.05) (Figure 3C).

#### 3.5.2 Peak expiratory flow

Three studies reported data on PEF (13, 20, 21). The meta-analysis showed no significant difference between the exercise and control groups (MD = 0.08, 95% CI: −0.31 to 0.47, I^2^: 0%, *P* = 0.70) (Figure 3D).

#### 3.5.3 Maximal expiratory and inspiratory pressures

Two studies (*n* = 32) (13, 20) assessed MEP and MIP. Pool results showed that exercise therapy significantly increased both MEP (MD = 10.70, 95% CI: 3.27 to 18.13, I^2^: 0%, *P* < 0.001) (Figure 3E) and MIP (MD = 16.44, 95% CI: 6.87 to 26.01, I^2^: 3%, *P* *<* 0.05) (Figure 3F).

#### 3.5.4 Six-minute walking test

Three studies involving 109 participants (13, 20, 22) measured functional exercise capacity using the 6MWT. No significant difference in exercise endurance was observed between the two groups (MD = 5.89, 95% CI: −14.75 to 26.53, I^2^: 52%, *P* = 0.58) (Figure 3G).

### 3.6 Subgroup analysis

Subgroup analysis was performed based on treatment cycle and single session duration (Table 4). Subgroup analyses revealed that an intervention frequency lasting 8 weeks significantly improved both FEV_1_ (MD = 0.31, 95%CI: 0.11 to 0.50, *P* = 0.002) and FVC (SMD = 0.43, 95% CI: 0.25–0.62, *P* < 0.001). Regarding session duration, interventions lasting 60 min or longer were associated with a significant increase in FEV_1_ (MD = 0.26, 95% CI: 0.09 to 0.44, *P* = 0.003), while a positive but non-significant trend was observed for FVC (SMD = 0.48, 95% CI: −0.01 to 0.98, *P* = 0.06).

**Table 4:** Results of subgroup analysis.

Subgroup	Group	Study	Heterogeneity test			Meta-analysis	
			*P*	*I^2^*	*Z*	MD/SMD (95% CI)	*P* value
**FEV_1_ (**Random effect, MD**)**							
Intervention frequency	≤8 w	1	-	-	-	0.28 [−0.01, 0.57]	0.06
	>8 w	3	0.25	28	3.07	0.31 [0.11, 0.50]	0.002**
Intervention time	<60 min	1	-	-	-	0.50 [0.08, 0.392]	0.02*
	≥60 min	3	0.42	0	2.93	0.26 [0.09, 0.44]	0.003**
**FVC (**Random effect, SMD**)**							
Intervention frequency	≤8 w	4	0.83	0	2.04	0.07 [0.00, 0.13]	0.04*
	>8 w	3	0.74	0	4.55	0.43 [0.25, 0.62]	<0.001***
Intervention time	<60 min	3	0.15	47	1.48	0.38 [−0.12, 0.88]	0.14
	≥60 min	4	0.09	54	1.91	0.48 [−0.01, 0.98]	0.06

*P < 0.05, **P < 0.01, ***P < 0.001.

### 3.7 Heterogeneity inspection and sensitivity analysis

Overall, the heterogeneity of most results is moderate to low (*I*^2^ values are 0% to 52%). For FEV₁/FVC, the meta-analysis revealed moderate heterogeneity (I² = 48%). Sensitivity analysis showed that after excluding the study by Kumar et al. (21), I² decreased to 0%, suggesting that this study was a major contributor to the observed heterogeneity. Similarly, the pooled result for the 6-minute walk test (6MWT) showed moderate heterogeneity (I² = 52%), with no statistically significant difference between groups. When the study by Yildirim et al. (20) was excluded, I² dropped to 0%, indicating that this study contributed substantially to the heterogeneity. In contrast, sensitivity analyses for FVC and FEV₁ demonstrated that the pooled effect sizes remained statistically significant after sequentially excluding individual studies, supporting the robustness of these findings.

## 4 Discussion

This systematic review and meta-analysis is the first to specifically evaluate the effects of exercise interventions on pulmonary function in adolescents with mild to moderate idiopathic scoliosis in short term. Although the included RCTs (*n* = 6) were generally of moderate to low quality, our findings indicate that exercise therapy significantly improves pulmonary function, including FEV_1_, FVC, FEV_1_/FVC, MIP, and MEP, compared to conservative management.

The diaphragm is attached to the ribs and lumbar vertebrae (24). Scoliosis, a three-dimensional spinal deformity, can impede rib movement through thoracic and lumbar curvature and rotation, thereby altering the length-tension relationship of the diaphragm, reducing thoracic compliance (7). In addition, there is a bidirectional interaction between chest wall compliance and respiratory muscle strength. Decreased chest wall compliance limits the depth of breathing, leading to reductions in MEP and MIP. This explains why even patients with mild AIS exhibit significantly lower respiratory function parameters than normal individuals (9), and those with larger spinal curvature demonstrate more pronounced abnormalities (25). Restrictive respiratory dysfunction is prevalent, observed in nearly two-thirds of patients with scoliosis (26), whereas obstructive and mixed ventilatory disorders are less common (8).

In patients with mild to moderate AIS, pulmonary function decline is often insidious but may adversely affect exercise tolerance, activities of daily living, and long-term quality of life. The findings of this meta-analysis demonstrate that exercise therapy significantly improves FEV₁ and FVC compared with conventional treatments, providing evidence-based support for its role in clinical rehabilitation. Our findings preliminarily confirm that significant improvements in FEV_1_ and FVC suggest exercise therapy can effectively mitigate the restrictive pulmonary pattern induced by AIS, a conclusion consistent with the results reported by Anthony et al. (14). FEV_1_ is an important indicator of large airway patency and expiratory muscle function, whereas FVC reflects maximum lung volume and serves as a key measure of restrictive ventilatory impairment. In patients with AIS, thoracic deformity and chest wall stiffness typically lead to mild-to-moderate reductions in both FEV_1_ and FVC. The observed improvements may be attributed to several mechanisms. First, active self-correction and thoracic mobility exercises incorporated into PSSE may improve chest wall compliance and increase expiratory driving pressure. Second, inspiratory muscle training can enhance respiratory muscle strength. Additionally, exercise therapy may indirectly improve FEV_1_ and FVC by enhancing overall physical fitness and respiratory coordination. Notably, although the included studies varied in intervention types, all demonstrated improvements in FEV_1_ and FVC. This finding suggests that different forms of exercise therapy may exert their effects through common or complementary physiological mechanisms.

In our meta-analysis, the pooled SMD for FEV_1_/FVC was 0.46 (95% CI: 0.05 to 0.86). This improvement does not represent a reversal of the restrictive pattern. Rather, it reflects optimization of expiratory function. The underlying mechanism is likely functional rather than structural. First, increased chest wall mobility, reducing small airway compression during forced exhalation. And it may improve cough efficiency, facilitating airway clearance and reduce airway resistance.

The intervention dosage is a crucial determinant of efficacy and must be carefully considered in clinical practice. This study provides a preliminary exploration of intervention duration and frequency. While subgroup analyses must be interpreted with caution due to limited statistical power, they offer insights for future research. Previous literature suggests that regular intervention cycles can help establish motor memory and consolidate neural adaptations (27). Longer-term interventions have demonstrated significant effects on improving the Cobb angle (28). An intervention cycle longer than 8 weeks may induce sustained physiological adaptations in respiratory muscle and thoracic mobility, which are critical for enhancing lung capacity. Although the current subgroup analysis did not reach statistical significance for the effect of intervention duration on FVC, the observed effect sizes suggest a potential trend favoring sessions longer than 60 min. A single session exceeding 60 min may provide sufficient time for adequate warm-up, technical training, and the necessary training volume to overload the respiratory system effectively, promoting strength and endurance gains. Shorter sessions may be insufficient to reach this critical threshold. Future research should not only confirm this dose-response relationship but also explore strategies to enhance efficiency and compliance, such as task-oriented approaches informed by ergonomics (29) or the integration of telerehabilitation technologies, to make effective high-dose interventions more feasible in the real world.

Although only two RCTs in our analysis reported MIP and MEP (13, 20), the substantial mean difference values (MIP: 16.44 cmH₂O; MEP: 10.70 cmH₂O) coupled with very low heterogeneity strongly indicate that exercise therapy can effectively enhance respiratory muscle strength. Exercise therapy includes core muscle group training, spinal adjustment, pulmonary function training, aerobic training, etc. Through repetitive task performance, these interventions can improve neuromuscular recruitment, thereby strengthening both inspiratory muscles (e.g., diaphragm, intercostal muscles) and expiratory muscles (e.g., abdominal muscles, intercostal muscles) (30), and enhance overall physical capacity (31).

Respiratory and skeletal muscle dysfunction are key factors limiting functional capacity in moderate AIS (32). We hypothesized that the physiological improvements in pulmonary volumes and respiratory muscle strength would translate into enhanced functional capacity and exercise tolerance. Fatigue and reduced exercise tolerance are common complaints among individuals with mild to moderate AIS (8). Targeted exercise and respiratory training could potentially improve quality of life and increase participation in physical activity. However, this review did not find a significant effect of exercise therapy on exercise endurance, as measured by the 6MWT, in patients with mild to moderate AIS. This null finding may be attributed to the limited number of included studies (*n* = 3). Furthermore, baseline imbalances, such as the slightly higher (though statistically non-significant) age, height, and weight in the control group of Yildirim’s study (20), which also reported higher baseline 6MWT values, might have influenced the pooled results. Exercise capacity was assessed using the 6-minute walk test (6MWT) in the included studies. While the 6MWT is a safe and clinically feasible measure of submaximal functional capacity, it may not fully capture improvements in peak exercise tolerance, particularly when interventions involve high-intensity exercise. Future studies should consider incorporating cardiopulmonary exercise testing to provide a more comprehensive assessment of exercise tolerance.

## 5 Limitations of the study

This systematic review and meta-analysis have certain limitations. First, the number of included RCTs is limited (*n* = 6), and the total sample size is relatively small (*n* = 224), which may lead to insufficient statistical power. Consequently, negative findings should be interpreted with caution, such as PEF and 6MWT. Second, considerable variability exists across the included studies in terms of exercise type, frequency, intensity, and intervention duration, which may explain the moderate heterogeneity observed in some outcomes (FEV_1_ and FVC) and affect the generalizability of the results. In this study, we attempted to perform subgroup analyses based on intervention types (e.g., PSSE, respiratory training, general exercise) to explore the effects of different interventions on the outcome measures. However, due to the limited number of included studies (a total of six), splitting the studies by intervention type resulted in insufficient sample sizes within each subgroup to meet the statistical power requirements for subgroup analysis. In addition, some intervention protocols exhibited content overlap (e.g., certain PSSE protocols also included respiratory training components), and forced categorization may have led to classification ambiguity and potential bias in the results. Therefore, we did not conduct formal subgroup analyses; instead, we pooled the data using a random-effects model and performed sensitivity analyses by sequentially excluding individual studies to account for the impact of heterogeneity on the pooled results. Third, pulmonary function in AIS is affected by factors such as height, weight, age, degree of scoliosis, and location of curvature (7). The direct comparison of pulmonary function tests before and after intervention may exaggerate the respiratory improvement after intervention. Therefore, more advanced evidence validation and the use of arm span standardized pulmonary function values are needed in the future (14). Fourth, none of the included studies provided long-term follow-up data, precluding any evaluation of the sustainability of the exercise interventions. Future research should focus on conducting larger, multicentric, high-quality randomized controlled trials to further clarify the optimal exercise protocols for patients with different curve characteristics (e.g., location of curvature). And conduct long-term follow-up to evaluate its long-term impact on the rate of pulmonary function decline, quality of life, and disease progression.

## 6 Conclusions

Exercise therapy may be an effective and safe intervention to improve respiratory muscle strength and pulmonary function in AIS patients. In clinical practice, rehabilitation programs should emphasize respiratory muscle training, enhancement of chest wall mobility, and improvement of overall physical fitness, rather than focusing on Cobb angle correction. Although the evidence quality of this literature is rated as moderate to low, the findings support the consideration of integrating structured exercise therapy into standardized management pathway for patients with AIS.

## Data Availability

The original contributions presented in the study are included in the article/Supplementary Material, further inquiries can be directed to the corresponding authors.
