# Supplementary material for: Short-term effects of exercise therapy on pulmonary function and exercise tolerance in patients with mild to moderate adolescent idiopathic scoliosis: a meta-analysis of randomized controlled trial
Source: Front Sports Act Living. 2026 Jun 17;8:1804651. doi: 10.3389/fspor.2026.1804651 (PMC13319063; doi:10.3389/fspor.2026.1804651)
Supplement: Supplementary file 2 [file Table1.pdf]

Supplementary Material 1. Detailed search strategy according to the PRISMA guideline.

Databases	Search Strategy
PubMed (n=82)	((((((((((((("Exercise Therapy"[Mesh]) OR (Rehabilitation Exercise[Title/Abstract])) OR (Exercise, Rehabilitation[Title/Abstract])) OR (Exercises, Rehabilitation[Title/Abstract])) OR (Rehabilitation Exercises[Title/Abstract])) OR (Therapy, Exercise[Title/Abstract])) OR (Exercise Therapies[Title/Abstract])) OR (Therapies, Exercise[Title/Abstract])) OR (Remedial Exercise[Title/Abstract])) OR (Exercise, Remedial[Title/Abstract])) OR (Exercises, Remedial[Title/Abstract])) OR (Remedial Exercises[Title/Abstract])) AND (("Scoliosis"[Mesh]) OR (Scolioses[Title/Abstract]))) AND ((("Randomized Controlled Trial" [Publication Type]) OR (randomized[Title/Abstract])) OR (placebo[Title/Abstract]))
Web of Science (n=181)	1: (TS=(scoliosis)) OR TS=(scolioses) and Preprint Citation Index<br>2: (((((((((((TS=(exercise therapy)) OR TS=(Rehabilitation Exercise)) OR TS=(Exercise, Rehabilitation)) OR TS=(Exercises, Rehabilitation)) OR TS=(Rehabilitation Exercises)) OR TS=(Therapy, Exercise)) OR TS=(Exercise Therapies)) OR TS=(Therapies, Exercise)) OR TS=(Remedial Exercise)) OR TS=(Exercise, Remedial)) OR TS=(Exercises, Remedial)) OR TS=(Remedial Exercises) and Preprint Citation Index<br>3: ((TS=(randomized controlled trial)) OR TS=(randomized)) OR TS=(placebo) and Preprint Citation Index<br>4: #1 AND #2 AND #3 and Preprint Citation Index
Scopus (n=251)	(TITLE-ABS-KEY (scoliosis OR "spinal curvature" OR "idiopathic scoliosis" OR "adolescent idiopathic scoliosis" OR kyphoscoliosis)) AND (TITLE-ABS-KEY ("exercise therapy" OR "physical therapy" OR "rehabilitation exercises" OR "therapeutic exercise" OR "Schroth method" OR "exercise intervention" OR "exercise program" OR "resistance training" OR physiotherapy)) AND (TITLE-ABS-KEY ("Randomized Controlled Trial" OR "randomized" OR "placebo"))
Embase (n=148)	#4. #1 AND #2 AND #3<br>#3. 'randomized controlled trial'/exp OR 'randomized':ab,ti OR 'placebo':ab,ti<br>#2. 'scoliosis'/exp OR 'scolioses':ab,ti<br>#1. 'exercise'/exp OR 'exercise therapy':ab,ti OR 'rehabilitation exercise':ab,ti OR 'exercise, rehabilitation':ab,ti OR 'exercises, rehabilitation':ab,ti OR 'rehabilitation exercises':ab,ti OR 'therapy, exercise':ab,ti OR 'exercise therapies':ab,ti OR 'therapies, exercise':ab,ti OR 'remedial exercise':ab,ti OR 'exercise, remedial':ab,ti OR 'exercises,

	remedial':ab,ti OR 'remedial exercises':ab,ti
The Cochrane Library (n=250)	#1. MeSH descriptor: [Scoliosis] explode all trees<br>#2. (Scoliosis):ti,ab,kw OR (Scolioses):ti,ab,kw<br>#3. #1 OR #2<br>#4. MeSH descriptor: [Exercise Therapy] explode all trees<br>#5. (Rehabilitation Exercise):ti,ab,kw OR (Exercise, Rehabilitation):ti,ab,kw OR (Exercises, Rehabilitation):ti,ab,kw OR (Rehabilitation Exercises):ti,ab,kw OR (Therapy, Exercise):ti,ab,kw OR (Exercise Therapies):ti,ab,kw OR (Therapies, Exercise):ti,ab,kw OR (Remedial Exercise):ti,ab,kw OR (Exercise, Remedial):ti,ab,kw OR (Exercises, Remedial):ti,ab,kw OR (Remedial Exercises):ti,ab,kw<br>#6. #4 OR #5<br>#7. #3 AND #6
