# Supplementary material for: Short-term effects of exercise therapy on pulmonary function and exercise tolerance in patients with mild to moderate adolescent idiopathic scoliosis: a meta-analysis of randomized controlled trial
Source: Front Sports Act Living. 2026 Jun 17;8:1804651. doi: 10.3389/fspor.2026.1804651 (PMC13319063; doi:10.3389/fspor.2026.1804651)
Supplement: Supplementary file 3 [file Table2.pdf]

Supplementary Material 2. Grade of evidence and effect estimates

Certainty assessment							№ of patients		Absolute effect (95% CI)	Certainty	Importance
**№ of studies**	**Study design**	**Risk of bias**	**Inconsistency**	**Indirectness**	**Imprecision**	**Other considerations**	**Experimental group**	**Control group**			
**Forced expiratory volume in 1 second (FEV1)**											
4	randomised trials	not serious	not serious	not serious	serious[b]	none	83	86	MD **0.41 L higher** (0.22 lower to 0.59 higher)	⊕⊕⊕◯ Moderate[b]	CRITICAL
**Forced vital capacity (FVC)**											
6	randomised trials	not serious	not serious	not serious	serious[b]	none	112	112	SMD **0.40 SD higher** (0.02 higher to 0.78 higher)	⊕⊕⊕◯ Moderate[b]	CRITICAL
**FEV1/FVC**											
5	randomised trials	not serious	not serious	not serious	serious[b]	none	95	95	SMD **0.46 SD higher** (0.05 higher to 0.86 higher)	⊕⊕⊕◯ Moderate[b]	CRITICAL
**Peak expiratory flow (PEF)**											
3	randomised trials	not serious	not serious	not serious	serious[b]	none	50	50	SMD **0.08 SD higher** (-0.31 lower to 0.47 higher)	⊕⊕⊕◯ Moderate[b]	IMPORTANT
**Maximal expiratory pressure (MEP)**											
2	randomised trials	not serious	not serious	not serious	serious[b]	none	32	32	MD **10.70 cmH2O higher** (3.27 higher to 18.13 higher)	⊕⊕⊕◯ Moderate[b]	IMPORTANT
**Maximal inspiratory pressure (MIP)**											
2	randomised trials	not serious	not serious	not serious	serious[b]	none	32	32	MD **16.44 cmH2O higher** (6.87 higher to 26.01 higher)	⊕⊕⊕◯ Moderate[b]	IMPORTANT
**Six minutes walking test (6MWT)**											
3	randomised trials	not serious	serious[a]	not serious	serious[b]	none	55	54	MD **5.89 higher** (-14.75 higher to 26.53 higher)	⊕⊕◯◯ Low[a,b]	IMPORTANT

GRADE interpretation: CI: confidence interval; **MD:** mean difference; **SMD:** standardised mean difference; a. The heterogeneity was large ($I^2 > 50\%$, representing potentially substantial heterogeneity); b. The total population size is less than 400, or there are less than 3 studies.
